# Mesonephric-Like Adenocarcinoma of the Endometrium: A Rare and Diagnostically Challenging Entity

**DOI:** 10.7759/cureus.110363

**Published:** 2026-06-06

**Authors:** Amresh Kirubahar, Sandhya Sundaram, Divya D, Barathi Gunabooshanam, Prithviraj PV

**Affiliations:** 1 Pathology, Sri Ramachandra Institute of Higher Education and Research, Chennai, IND; 2 Surgical Oncology, Sri Ramachandra Institute of Higher Education and Research, Chennai, IND

**Keywords:** endometrial carcinoma, gata3, immunohistochemistry, mesonephric-like adenocarcinoma, pax8, postmenopausal bleeding, rare tumour

## Abstract

Mesonephric-like adenocarcinoma (MLA) is a recently recognized and rare entity. It is a morphologically distinctive carcinoma of the uterine corpus, estimated to account for approximately 1% of endometrial malignancies. Its diagnosis is particularly challenging owing to morphological and immunophenotypic overlap with cervical mesonephric adenocarcinoma and other endometrial carcinoma subtypes. Accurate identification has significant clinical implications given its association with aggressive behavior. We report the case of a 62-year-old postmenopausal woman who presented with a two-day history of postmenopausal vaginal bleeding.

A PET/MR fusion scan revealed a metabolically active, T2-hyperintense lesion involving the posterior uterine wall and lower uterine segment, associated with haematometra. She underwent a total abdominal hysterectomy with bilateral salpingo-oophorectomy and comprehensive surgical staging. Histopathological examination demonstrated a high-grade carcinoma with tumor cells arranged in small tubules, nests, and sheets, containing intraluminal eosinophilic secretions, a pattern characteristic of mesonephric-like differentiation. Immunohistochemistry showed diffuse positivity for GATA3 and PAX8, with wild-type p53 expression, supporting the diagnosis of MLA. Tumor invasion exceeded 50% of the myometrial thickness (93.75%), with negative lymph nodes and clear surgical margins, yielding a final stage of pT1b pN0 (the International Federation of Gynecology and Obstetrics (FIGO) stage IIC).

This report underscores the critical importance of integrating classical morphological assessment with a targeted immunohistochemical panel in diagnosing MLA. As this entity is increasingly recognized, pathologists must maintain a high index of suspicion, particularly in postmenopausal women with tumors exhibiting tubular architecture and eosinophilic luminal secretions.

## Introduction

Mesonephric adenocarcinoma (MA) is a well-established but rare gynecologic tumor, classically arising in the uterine cervix from embryological remnants of the mesonephric (Wolffian) duct. It is associated with a notoriously aggressive clinical course and poses a considerable diagnostic challenge for both clinicians and pathologists [1,2,3]. In recent years, tumors with nearly identical morphology and immunophenotype have been reported in sites where mesonephric remnants are not typically present, most notably the uterine corpus and, to a lesser extent, the ovary. Recognizing this, the 2020 WHO Classification of Female Genital Tumors formally introduced the designation “mesonephric-like adenocarcinoma” (MLA) as a distinct entity [4]. This nomenclature deliberately acknowledges the histological and molecular resemblance to true MA, while highlighting that its pathogenesis may differ fundamentally.

From an immunohistochemical standpoint, MLA closely resembles MA, typically expressing GATA3, TTF1, PAX8, and CD10, while being consistently negative for estrogen and progesterone receptors (ER/PR) [5,6]. Molecularly, both tumor types harbor KRAS mutations and chromosome 1q gain; however, MLA more frequently demonstrates PIK3CA and PTEN alterations, aligning it more closely with conventional endometrial endometrioid carcinoma [7,8,9]. These findings have led some investigators to propose that MLA may represent a Müllerian-derived tumor undergoing mesonephric-like transdifferentiation, potentially in the context of associated endometriosis, though its precise histogenesis remains an active area of investigation [10,11].

Clinically, MLA has been associated with aggressive biological behavior, with a significant proportion of patients presenting at an advanced stage or experiencing recurrence despite apparently complete surgical resection [12]. Given its rarity, accounting for approximately 1% of endometrial carcinomas, it remains largely unfamiliar in routine surgical pathology practice, and misclassification is not uncommon [13]. We present a case of MLA arising in the uterine corpus of a 62-year-old postmenopausal woman. This report aims to highlight the characteristic morphological and immunohistochemical features of this entity and to emphasize the clinical importance of accurate recognition.

## Case presentation

Clinical history and imaging

A 62-year-old postmenopausal woman presented to the gynecology clinic with a two-day history of vaginal bleeding. She had attained natural menopause approximately 10 years earlier and had no significant gynecological history. There was no family history of gynecological or colorectal malignancy.

A PET/MR fusion scan demonstrated a metabolically active, heterogeneously enhancing lesion involving the cervix, with no evidence of metabolically active retroperitoneal lymphadenopathy or distant metastatic disease. The lesion appeared as a T2-hyperintense lesion with significant restricted diffusion (Figure 1). The tumor predominantly involved the posterior uterine wall and the lower uterine segment, resulting in narrowing of the cervical canal. The endometrial cavity was distended and filled with fluid, and a T2-hypointense clot was present within it (Figure 2). Minimal parametrial stranding was noted along the left lateral wall. A few subcentimeter bilateral external iliac lymph nodes with minimal metabolic activity were identified, though these were not considered pathologically significant.

**Figure 1:**
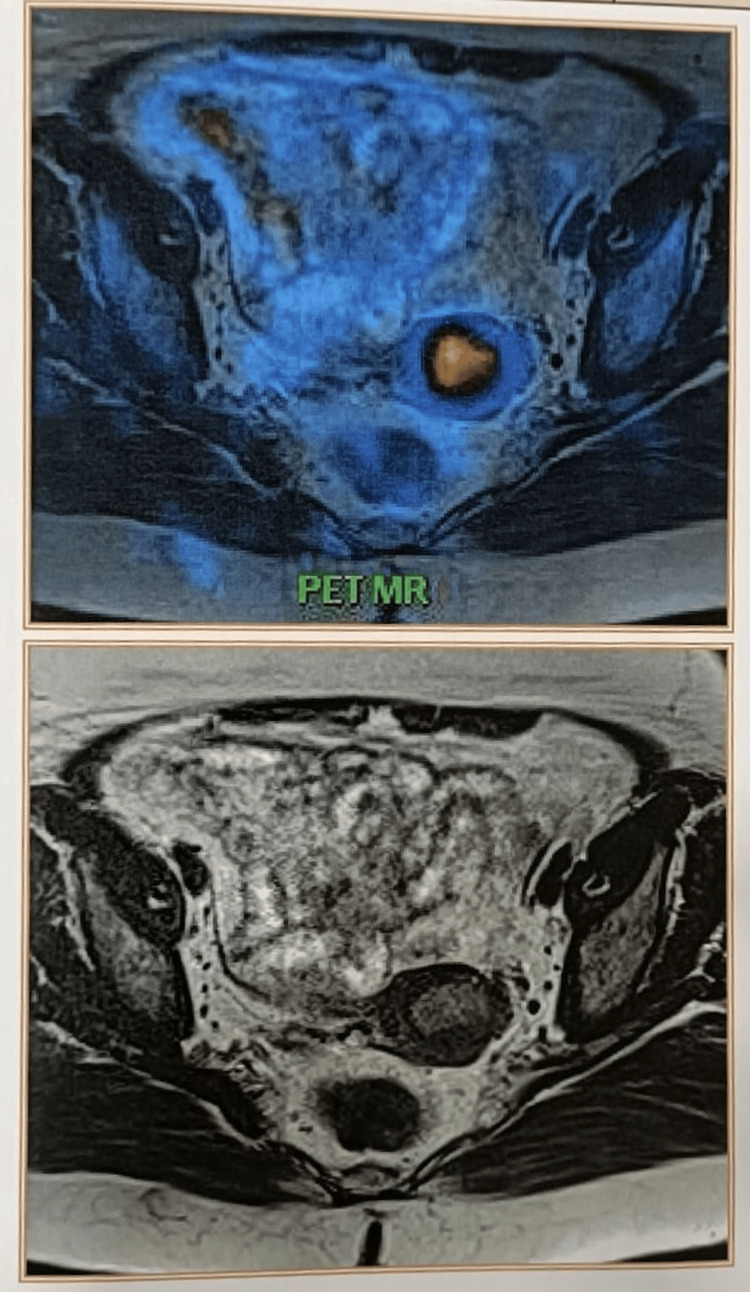
Axial PET/MR fusion images The images show an FDG-avid heterogeneously enhancing cervical lesion involving the posterior wall and lower uterine segment with cervical canal narrowing (SUVmax 3.3). The lesion appears T2 hyperintense with restricted diffusion PET: positron emission tomography; MR: magnetic resonance; FDG: fluorodeoxyglucose; SUVmax: maximum standardized uptake value

**Figure 2:**
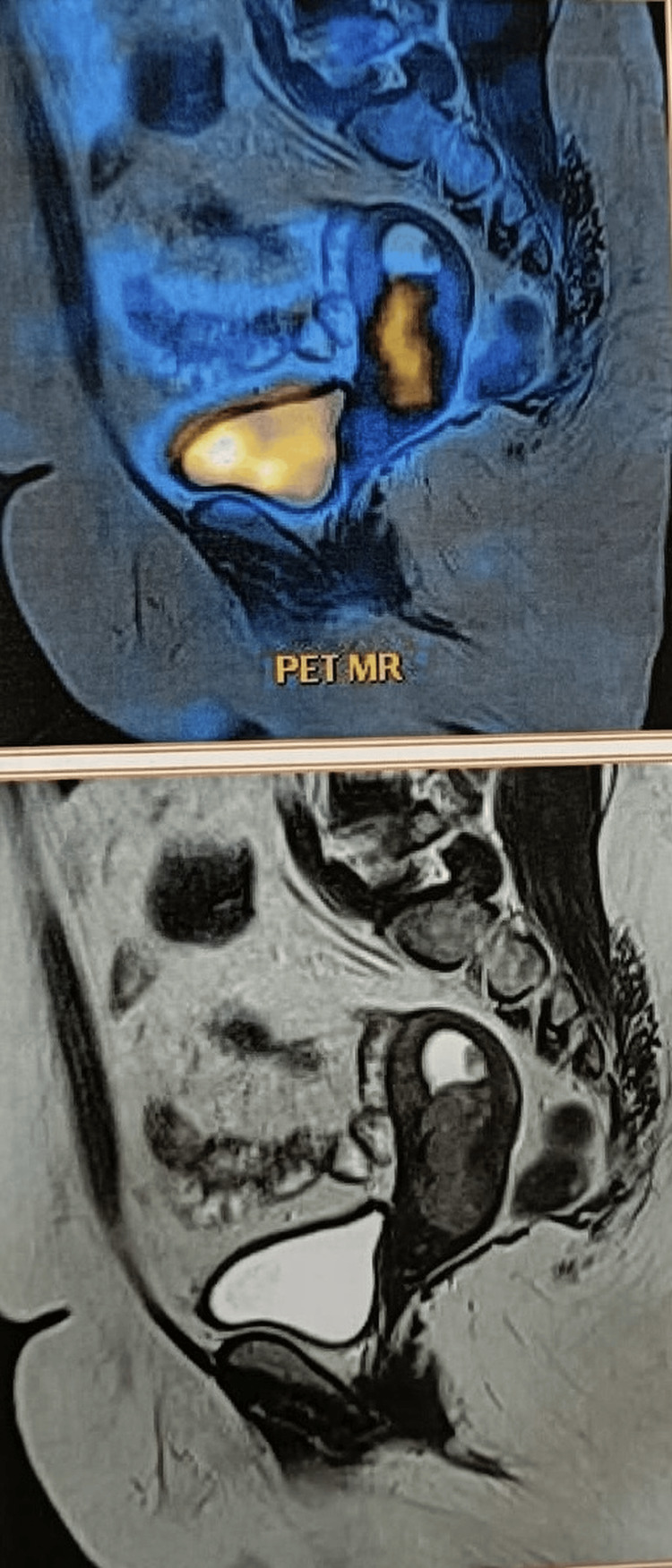
Sagittal PET/MR fusion images The images demonstrate a metabolically active cervical mass with extension into the lower uterine segment, associated with endometrial cavity distension containing fluid and clot. No gross parametrial invasion is identified PET: positron emission tomography; MR: magnetic resonance

Following a multidisciplinary review, the patient was taken to theatre and underwent a total abdominal hysterectomy with bilateral salpingo-oophorectomy (TAH-BSO) and comprehensive staging laparotomy. During intraoperative assessment, a small liver surface nodule was incidentally identified and sampled for histopathological examination to rule out metastatic disease. Surgical specimens submitted for histopathological analysis included the uterus with cervix, bilateral adnexae, right and left pelvic lymph nodes, omentum, para-aortic lymph nodes, and the liver surface nodule.

Pathological evaluation

Gross Findings

On gross examination, the uterus harbored a firm, solid, grayish-white tumor with focal friable areas, involving the lower uterine segment and extending toward the endocervical canal. The total myometrial thickness was measured at 1.6 cm. The estimated depth of myometrial invasion was 1.5 cm, representing more than 50% invasion of the myometrial wall. The distance to the left and right parametrial margins was both 1.2 cm, and the vaginal cuff margin measured 0.6 cm. The bilateral fallopian tubes and ovaries appeared grossly unremarkable. The uninvolved endometrium extended 2 cm from the cervix.

Microscopic Findings

Microscopic examination revealed a high-grade carcinoma with a striking tubular architecture. Tumor cells were arranged predominantly in small, well-defined tubules, with focal solid sheets and nests (Figure 3). Many tubules contained characteristic intraluminal eosinophilic secretions, which are a hallmark feature of mesonephric differentiation. The individual tumor cells were cuboidal to columnar, with vesicular nuclear chromatin, moderate nuclear pleomorphism, and moderate to scant cytoplasm (Figure 4). Focal areas of necrosis and hemorrhage were noted.

**Figure 3:**
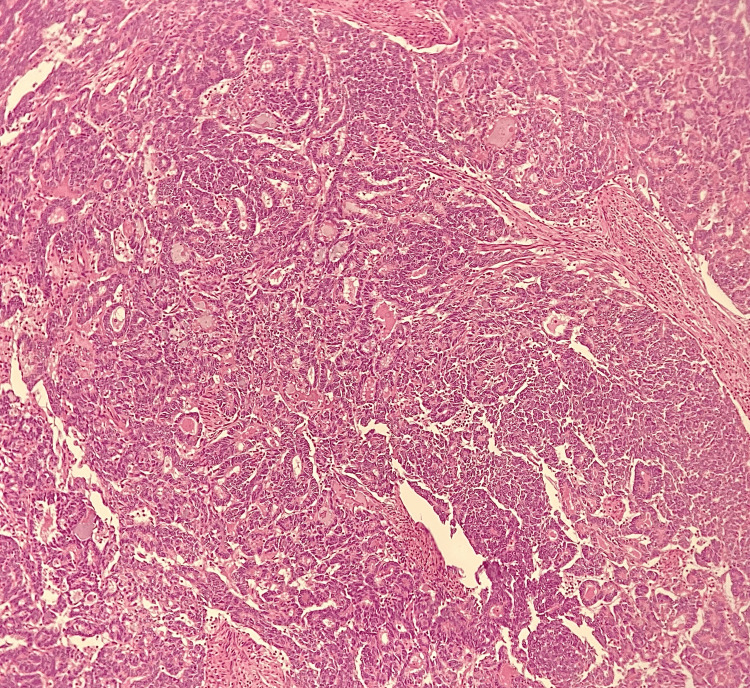
Low-power H&E-stained photomicrograph The image demonstrates endometrial tissue with a malignant infiltrating neoplasm composed of tumor cells arranged in small tubules, sheets, and nests. Many tubules show intraluminal secretions (40x)

**Figure 4:**
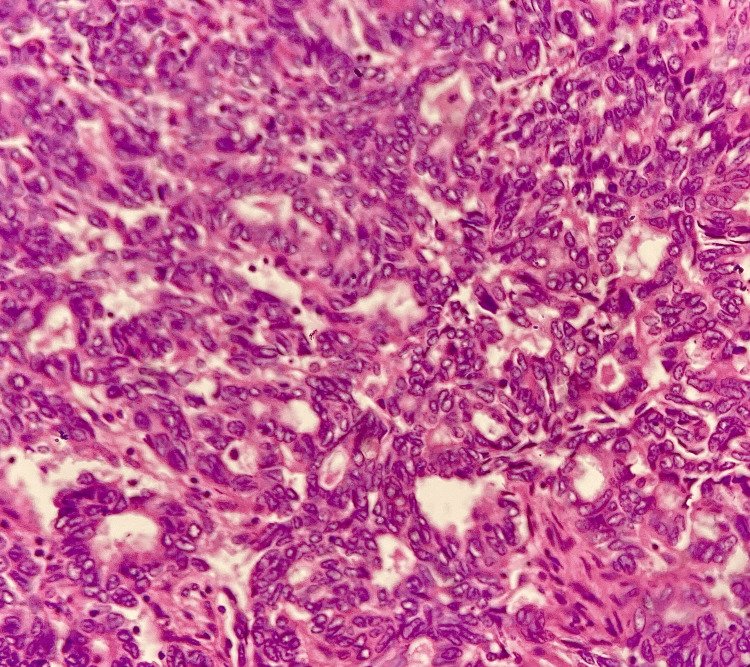
H&E-stained photomicrograph The image shows tumor cells with increased nuclear-cytoplasmic ratio, moderate nuclear pleomorphism, and vesicular chromatin (100x)

Myometrial invasion was confirmed, with a depth of 1.5 cm corresponding to 93.75% of the myometrial thickness, indicating deeply invasive disease. Adenomyosis was not identified. The cervix was extensively sampled, and no cervical stromal invasion was identified. Lymphovascular invasion was absent, and all surgical margins were free of invasive carcinoma. All 44 regional lymph nodes were examined, and none showed evidence of metastatic disease. The peritoneal wash cytology was negative for malignant cells. The liver surface nodule showed unremarkable hepatic parenchyma with proliferating biliary radicles, free of tumor. The omentum was uninvolved. The bilateral fallopian tubes showed hydrosalpinx. Both ovaries demonstrated normal physiological changes.

The pathological TNM staging was pT1b (tumor invading one-half or more of the myometrium), pN0 (no regional lymph node metastasis), the International Federation of Gynecology and Obstetrics (FIGO) stage IIC (aggressive histological type with myometrial involvement).

Immunohistochemical Analysis

An immunohistochemical panel was applied to confirm the nature of the tumor and to exclude morphological mimics. Tumor cells demonstrated diffuse nuclear positivity for GATA3 (Figure 5) and PAX8 (Figure 6). p53 expression was wild-type (patchy, heterogeneous staining), effectively excluding a p53-aberrant (serous-type) carcinoma. Cyclin D1 was negative. p16 staining was non-contributory. The overall immunophenotype - particularly the combination of GATA3 positivity with PAX8 expression, absence of ER and PR expression, and the characteristic tubular morphology with eosinophilic secretions - was diagnostic of mesonephric-like adenocarcinoma (Table 1).

**Figure 5:**
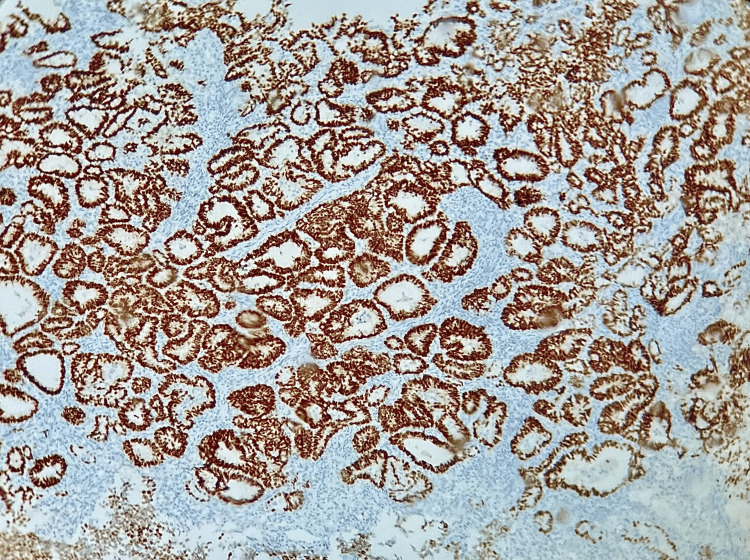
GATA3 immunohistochemical stain demonstrating diffuse nuclear positivity in tumor cells (100x)

**Figure 6:**
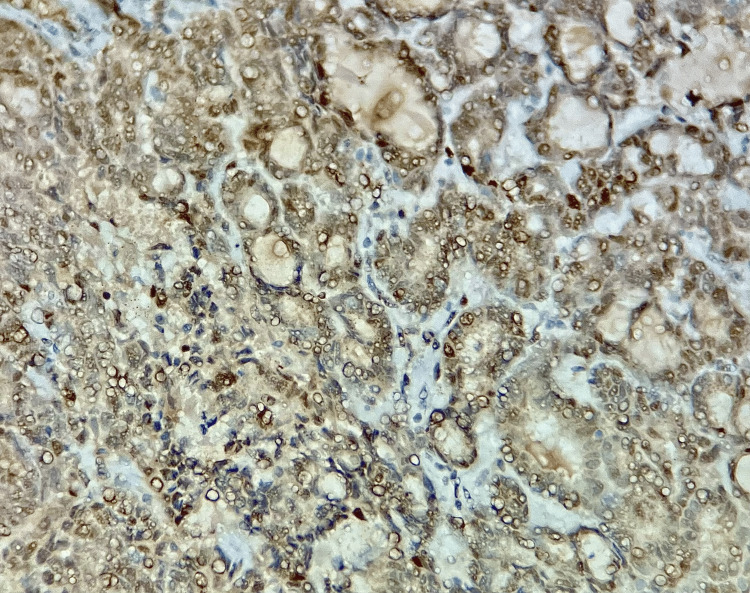
PAX8 immunohistochemical stain demonstrating diffuse nuclear positivity in tumor cells (200x)

**Table 1:** Summary of immunohistochemical results ER: estrogen receptor; PR: progesterone receptor; MLA: mesonephric-like adenocarcinoma

Marker	Result	Interpretation
GATA3	Diffuse positive (+3)	Supports mesonephric differentiation
PAX8	Diffuse positive	Müllerian/mesonephric origin
p53	Wild-type (patchy)	Excludes TP53-aberrant carcinoma
Cyclin D1	Negative	Against endometrioid carcinoma
p16	Non-contributory	—
ER/PR	Negative (by implication)	Supports MLA over endometrioid

## Discussion

This case illustrates the diagnostic complexity inherent to MLA, an entity that sits at the intersection of multiple well-known gynecological tumor types. Arriving at the correct diagnosis required a systematic integration of clinical context, morphological features, and a carefully tailored immunohistochemical panel. From the outset, the clinical presentation was somewhat atypical. Postmenopausal bleeding in a 62-year-old woman, in isolation, would raise the suspicion of the far more common endometrioid endometrial carcinoma. However, the imaging profile - particularly the metabolically active cervical lesion on PET-CT and the deeply invasive T2-hyperintense mass on MRI - hinted at a more aggressive process, prompting thorough surgical staging.

Histologically, the architectural pattern was the first and most important diagnostic clue. The tumor was composed predominantly of small, back-to-back tubules - many harboring the characteristic intraluminal eosinophilic secretions that have become synonymous with mesonephric differentiation. Alongside this, focal solid and nested growth patterns were observed, and cytologically the cells were cuboidal to columnar with vesicular chromatin, features well-described in published series of MLA [1,13]. The absence of squamous differentiation, mucinous change, or the typical glandular complexity of endometrioid carcinoma made that diagnosis untenable. Similarly, the absence of significant papillary architecture and the wild-type p53 pattern effectively excluded high-grade serous carcinoma - a crucial distinction given the implications for adjuvant treatment [7].

The immunohistochemical profile was confirmatory. Diffuse GATA3 positivity is perhaps the most important marker in this context; while GATA3 is routinely used in breast and urothelial pathology, its expression in gynecological tumors has emerged as a reliable indicator of mesonephric (and mesonephric-like) differentiation [9,11]. Concurrent PAX8 positivity, as seen in this case, reflects a Müllerian-related or renal tubular origin and is expected in MLA. The tumor showed negative expression for ER and PR. The negative cyclin D1 helped to further distance the tumor from endometrioid carcinoma, where cyclin D1 overexpression is frequently encountered. Wild-type p53 was an important finding, as aberrant p53 (either diffuse or completely absent) would have redirected the diagnosis toward serous carcinoma or FIGO grade 3 endometrioid carcinoma with p53 mutation.

The depth of myometrial invasion in this case (93.75%) is striking and merits emphasis. Despite this profoundly invasive growth, all 44 lymph nodes were free of metastasis, peritoneal cytology was negative, and all margins were clear. This apparent paradox between depth of invasion and the absence of nodal spread has been noted in prior series of MLA and may reflect the relatively early stage at which the patient presented, perhaps facilitated by the symptom of postmenopausal bleeding prompting timely clinical attendance [13]. The pathological staging of pT1b pN0 (FIGO IIC) places this patient in a category that would typically warrant adjuvant treatment, and the designation as an "aggressive histological type" under the FIGO 2023 classification system is clinically meaningful, as it confers a worse prognosis than stage-matched conventional endometrioid carcinoma [7].

From a broader perspective, MLA remains an under-recognized entity. Its rarity means that many pathologists may encounter it only once or twice in a career, and unless a deliberate effort is made to include it in the differential diagnosis of unusual endometrial tumors, it may be misclassified as high-grade endometrioid, serous, or even clear cell carcinoma. The consequences of misclassification are not trivial, as MLA has distinct molecular alterations (KRAS, PIK3CA, PTEN, 1q gain) that are increasingly relevant in the era of molecular-guided therapy, and accurate classification enables appropriate enrollment into clinical trials or targeted treatment strategies [8,12].

We advocate for a low threshold in applying GATA3 immunohistochemistry when confronted with an endometrial carcinoma that does not fit neatly into the conventional categories, particularly when a tubular growth pattern with eosinophilic secretions is observed.

## Conclusions

MLA of the endometrium is a rare but clinically significant entity that demands careful morphological assessment supported by a judiciously chosen immunohistochemical panel. This case report - involving a 62-year-old postmenopausal woman with deeply invasive endometrial carcinoma displaying tubular architecture, luminal eosinophilic secretions, GATA3 positivity, and wild-type p53 - exemplifies the diagnostic approach required to confidently establish this diagnosis. Accurate identification of MLA is essential not only for prognostication but increasingly for molecular characterization and the selection of targeted therapies. As awareness of this entity grows within the pathology community, its recognition in routine practice will improve, ultimately benefiting patients through more precise and personalized management.
